# Diagnostic Performance and Appropriate Cut-Offs of Different Anthropometric Indicators for Detecting Children with Overweight and Obesity

**DOI:** 10.1155/2021/1608760

**Published:** 2021-09-15

**Authors:** Muhammad Asif, Muhammad Aslam, Kalim Ullah, Muhammad Qasim, Khurram Afzal, Asad Abbas, Manzar Ali, Muhammad Younis, Sami Ullah, Muhammad Hassham Hassan Bin Asad, Justyna Wyszyńska

**Affiliations:** ^1^Department of Statistics, Government Degree College, Qadir Pur Raan, Multan, Punjab, Pakistan; ^2^Department of Statistics, Bahauddin Zakariya University, Multan, Pakistan; ^3^Department of Zoology, Kohat University of Science and Technology (KUST), Kohat, Pakistan; ^4^Department of Economics, Finance and Statistics, Jonkoping University, Sweden; ^5^Institute of Food Science & Nutrition, Bahauddin Zakariya University, Multan, Pakistan; ^6^Multan Medical & Dental College, Ibne Siena Hospital & Research Institute, Multan, Pakistan; ^7^Department of Pharmacy, University of Peshawar, KPK, Pakistan; ^8^Department of Pharmacy, COMSATS University, Islamabad, Abbotabad Campus, 22060, Pakistan; ^9^Institute of Fundamental Medicine, Department of Genetics, Kazan Federal University, Kazan, Russia; ^10^Medical College of Rzeszów University, ul. Kopisto 2a, 35-959 Rzeszów, Poland

## Abstract

In the clinical settings, different anthropometric indicators like neck circumference (NC), waist circumference (WC), midupper arm circumference (MUAC), waist-to-height ratio (WHtR), and arm-to-height ratio (AHtR) have been suggested for evaluating overweight and obesity in children. The comparative ability of these indicators in Pakistan is yet unknown. This study is aimed at examining the validity of different anthropometric indicators of overweight and obesity simultaneously and at determining their superlative cut-off values that would correctly detect overweight and obesity in children. For this purpose, the dataset of anthropometric measurements height, weight, WC, MUAC, and NC of 5,964 Pakistani children, aged 5-12 years collected in a cross-sectional multiethnic anthropometric survey (MEAS), was used. Receiver operating characteristic (ROC) curve analysis was performed to assess the validity of different anthropometric indicators. The most sensitive and specific cut-off points, positive and negative predictive values of each indicator were also calculated. The results of the ROC curve indicated that all the studied indicators had a good performance but the indicators AHtR and WHtR had the highest value of the area under the curve (AUC) for the screening of children with overweight and obesity (AUC > 0.80). In the overall sample, AHtR, WHtR, MUAC, WC, and NC cut-off points indicative of overweight, in both boys and girls, were 0.14, 0.46, 18.41 cm, 62.86 cm, and 26.36 cm and 0.14, 0.47, 18.16 cm, 64.39 cm, and 26.54 cm, respectively; the corresponding values for obesity were 0.14, 0.47, 18.67 cm, 62.10 cm, and 26.36 cm and 0.14, 0.48, 20.19 cm, 64.39 cm, and 25.27 cm. We concluded that the sex-specific cut-off points for AHtR, WHtR, MUAC, WC, and NC can be used to diagnose overweight and obesity in Pakistani children.

## 1. Introduction

Childhood obesity has now become a grave public health issue, and its prevalence over the past years has risen dramatically worldwide [1]. Various established factors including socioeconomic development, sedentary habits, consumption of imbalance, high caloric, proteinase, and fat-containing diets, and lack of physical activity may cause the development of obesity and its related metabolic complications [2–4]. Epidemiological evidence consistently reports that obesity also concerns to the Pakistani children [5–7]. A cross-sectional study with the Pakistani children has shown that the prevalence of overweight and obesity in Pakistani primary school children was about 17% and 7.5%, respectively [3]. These results were a four-time increase in overweight school-aged children in the past five years.

Researchers used different methods in the preliminary diagnosis of obesity and its consequences [8]. But anthropometry, whose concerned with the measurement of physical sizes, was considered as an internationally acceptable method that can be easily applied to a large population. Body mass index (BMI) is the most extensively used criteria for defining overweight and obesity in both children and adults [1]. Growing evidences from specific populations showed that body fat deposition enormously fat in the central (abdominal) and upper body segment is a better predictor of some obesity-related complications, such as hypertension, diabetes, and heart disease [9]. Therefore, different studies utilized different anthropometric indices, i.e., waist circumference (WC), waist-to-height ratio (WHtR), and neck circumference (NC) as an index to determine regional [10–13]. Few investigators in recent years also evaluated the diagnostic performance of midupper arm circumference (MUAC) and arm-to-height ratio (AHtR) and proposed that both of these indicators are simple, inexpensive, and accurate measures for obesity screening in children [14–17].

All the above-mentioned anthropometric indicators are effective, are more practical, and have been used for obesity screening purposes. However, the investigation about the efficacy of different anthropometric indicators for screening children with overweight and obesity still remains scarce and needs to be evaluated in Pakistan. There was also a need to determine the optimal cut-offs of children in Pakistan. Given this negotiable need, we planned the present study.

The goals of the present research were as follows: (1) to determine whether all the studied anthropometric indicators are equally efficient for diagnosing overweight and obesity in Pakistani children as measured by BMI and (2) to determine their superlative cut-off values that would correctly detect overweight and obesity in children.

## 2. Materials and Methods

The study used secondary data, collected in a cross-sectional multiethnic anthropometric survey (MEAS) that was carried out from March through June 2016 in the 4 populated cities of Pakistan, viz., Lahore, Islamabad, Rawalpindi, and Multan. The data was also publicly available in Mendeley (https://data.mendeley.com/datasets/sxgymx5xjm/1). Detailed description about the study design and the sampling methodology in this survey was described elsewhere [18–21]. Briefly, in the MEAS, a total of 10,782 children and adolescents (aged 2-19 years) were recruited and the dataset of school-going children and adolescents (*n* = 9,929) aged 5 to 19 years were collected from 68 public and private schools. The school (s) selection and subject's selection were totally based on probability sampling, while the data for below 5-year-aged subjects were gathered from public places (i.e., markets, shopping malls, and parks) using convenient sampling. As the basic purpose of MEAS was to construct the sex- and age-specific anthropometric growth reference charts, therefore, data of this survey already have been used for the construction of new WC, WHtR, and BMI percentile curves for the Pakistani children [20, 21]. For this investigation, we have only included 5,964 subjects aged 5 to 12 years to make the diagnostic performance comparison of newly proposed anthropometric indicators (i.e., MUAC, WC, NC, AHtR, and WHtR) of overweight and obesity using receiver operating characteristic (ROC) analysis. The children were excluded in this survey, if they were having some chronic diseases that may affect the body composition (e.g., diabetes, metabolic syndromes, hyperlipidemia, or thyroid diseases). As this study used secondary data, children's or guardian's informed consent was not required. In addition, the authors assert that the complete study protocol was according to the ethical standards declared by Helsinki.

In this survey, the raw dataset of different anthropometric characteristics, i.e., body weight, height, NC, WC, and MUAC, was taken in a comfortable standing position under standard procedure [18–21]. From the body measurements, different indices were calculated: body mass index (BMI = weight (kg.) ÷ height (meters)^2^); waist-to-height ratio (WHtR = waist circumference (cm) ÷ height (cm)); arm-to-height ratio (AHtR = midupper arm circumference (cm) ÷ height (cm)). The MEAS was approved by the Institutional Ethics Research Board of Bahauddin Zakariya University, Multan, under the registration number IRB# Stat-271/2017.

### 2.1. Statistical Analysis

In this study, overweight and obesity in children were defined by using BMI. As in Pakistan, nation-based reference data of BMI were not available. We therefore used age- and sex-specific BMI reference values from the World Health Organization (WHO) as a cut-off point [22]. An individual was considered to be overweight if 85 < BMI ≤ 95^th^ percentile and obese if BMI > 95^th^ percentile.

The statistical analysis was performed in software “Statistical Package for Social Sciences (SPSS)” version 24. 0. Initially, the descriptive statistics of each anthropometric variable in the form of mean ± standard deviation (SD), median (interquartile range (IQR)) are presented. Average significant differences between boys and girls were checked by using the *t*-test. The multiple linear regression and Pearson correlation coefficient (*r*) were used to examine the relationship between BMI and other anthropometric indicators. Receiver operating characteristic (ROC) curve analysis was applied for checking the predictive validity and determination of cut-off points of each anthropometric indicator for identifying children with overweight and obesity. Different characteristics of ROC curve analysis, i.e., area under the curve (AUC) along with 95% confidence interval (CI), sensitivity (true-positive rate), and specificity (true-negative rate), were presented. Following the Perkins and Schisterman guidelines [23], AUC values were interpreted as follows: if an anthropometric indicator has an AUC between 0.65 and 1.00, then the test is considered to be “highly accurate,” and if an anthropometric indicator has an AUC between 0.50 and 0.65, then the test is considered to be “moderately accurate.” The AUC = 0.5 indicating that the screening test is no better than chance, i.e., noninformative. The positive predictive value (PPV), positive likelihood ratio (*L*_P_), negative predicted value (NPV), and negative likelihood ratio (*L*_N_) for each indicator were also computed by sex [10]. The positive predicted value represents the proportion of subjects who have a disease (overweight/obesity) and have a positive test. The negative predicted value is the proportion of subjects without a disease (i.e., normal weight) and has a negative test. The same ROC curve analysis was also performed for prepubertal and pubertal-aged children. The significance level was set at *α* = 5% for the whole analysis.

## 3. Results

A total of 5,964 children (boys = 2,865 and girls = 3,099) were included in the study with mean age of 8.87 (±2.36) years. The classification of nutritional status using WHO BMI reference values showed that 11.7% of children were overweight and 4.7% were obese. Overweight and obesity prevalence in boys were 11.2% and 5.0% and in girls were 12.2% and 4.4%, respectively. The descriptive statistics of age and anthropometric characteristics by sex are also described in Table 1.

The average values of age and other anthropometric characteristics (height, weight, NC, WC, and MUAC) were significantly higher in boys as compared to girls, while the average values of BMI and WHtR were not significantly different among the children of both sexes. Results of the correlation and regression analysis, examining the relationship between BMI and other obesity indicators, are presented in Tables 2(a) and 2(b).

In each sex and the overall sample, significant (*p* < 0.001) positive correlations between BMI and all other proxy measures of obesity were found. In the overall sample, the significant positive correlations were observed between BMI and MUAC (*r* = 0.65), followed by NC (*r* = 0.56), WC (*r* = 0.56), AHtR (*r* = 0.54), and WHtR (*r* = 0.44). Regression analysis also revealed that about 52.0% variation in BMI is explained due to the predictor variables including the age and sex.

**Figure 1:**
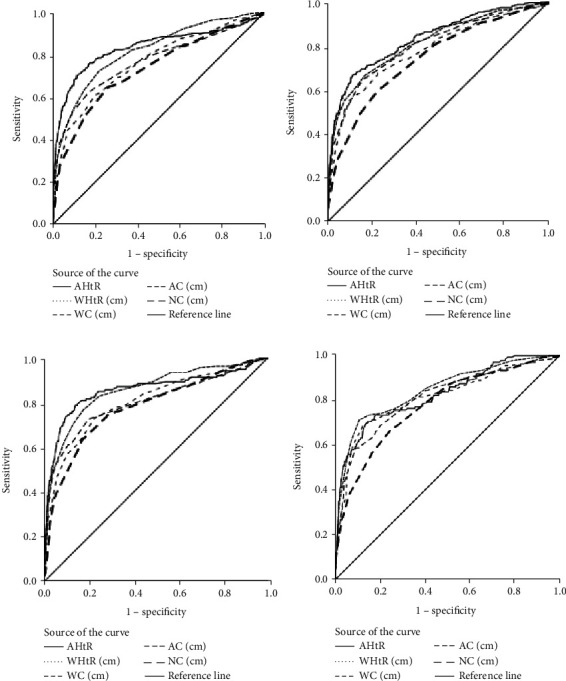
Receiver operating characteristic (ROC) curves of anthropometric parameters as an indicator of overweight ((a) boys; (b) girls) and obesity ((c) boys; (d) girls).

Figure 1 illustrates the accuracy of individual indicators in identifying children with overweight and obesity by ROC curves, and Table 3 presents the information about the AUC of the curves among Pakistani children.

In an overall sample of both sexes, AUC results reveal that all proxy indicators had a “highly accurate” performance (i.e., AUC > 0.65) for screening children with overweight and obesity (AUC range: 0.73 to 0.86; 95% CI: (0.700-0.900)). However, anthropometric indicators of AHtR and WHtR had more predictive abilities in identifying overweight and obesity than others. The cut-off points, sensitivities, specificities, PPV, NPV, *L*_P_, and *L*_N_ of each proxy indicator in diagnosing overweight and obesity are shown in Table 4.

For the overall children sample, the most sensitive and most specific AHtR cut-off values for defining overweight and obesity were identical in both boys and girls, i.e., 0.14. The suggested cut-off values of WHtR were, respectively, 0.46 and 0.47 for defining overweight and obese boys and 0.47 and 0.49 for girls. The cut-off values of WC in girls (64.39 cm and 68.29 cm) were higher than in boys (62.86 cm and 64.84 cm) for both excess body mass categories. Similarly, the cut-off values of MUAC and NC in girls were also higher than in boys for defining obesity (for MUAC: girls vs. boys: 20.19 cm vs. 18.67 cm, respectively, and for NC: girls vs. boys: 27.52 cm vs. 27.05 cm, respectively). The likelihood ratios for each cut-off point were also shown. For example, a girl with AHtR > 0.14 indicated that she is 4.74 times more likely to be obese (i.e., BMI > 95^th^ percentile) than a girl with an AHtR value below this cut point.

## 4. Discussion

Obesity in children is now considered to be a serious chronic health issue, affecting both developed and developing countries [24, 25]. In the last two decades, Pakistani children have seen the marked increases in the prevalence of overweight and obesity [5, 6]. Appropriate early-stage diagnosis and treatment of obesity in childhood are important priorities of health practitioners for reducing the obesity-related disorders in adulthood [24]. Different practical indices are applicable to diagnosing obesity and its associated metabolic risks. A systematic review and meta-analysis by Alves et al. [26] indicated that BMI, WC, and WHtR had high discriminatory power (AUC > 0.897) to identify body fat in children and adolescents. In recent years, few reports also utilized some other anthropometric indices (i.e., MUAC, AHtR, and NC) as an index to determine regional adiposity [10, 14–17].

To date, different epidemiological researchers proposed different cut-off values of these indicators for determining obesity in children and adolescents [11, 13]. Generally, WC 90^th^ percentile and WHtR 85^th^ percentile are often used as clinical cut-off points for abdominal obesity and its related risks [27–29]. Fujita et al. [12] calculated the WHtR cut-off points of 0.51 for Japanese school-age boys and 0.50 for girls to classify abdominal adiposity using ROC curve analysis (9-11 years). Another research conducted by Sousa et al. found the threshold value of WHtR identified in both boys and girls to be 0.45 [30].

For MUAC, Lu et al. [16] provided the age- and sex-specific cut-off points (ranged between 18.9 and 23.4 cm) to identify Han children with elevated BMI (BMI > 85^th^ percentile). They also proposed a single age- and sex-independent cut-off of AHtR > 0.15 for identifying children with overweight and obesity. Currently, Nafiu et al. [10] propose that NC could be used to identify children who are overweight and obese. A Canadian researcher Katz et al. [31] developed the reference data of NC, and they found that NC above the 50^th^ centile is a responsive predictor of overweight and obesity (BMI > 85^th^ centile). According to one study, the cut-off values for NC that demonstrated a higher sensitivity and specificity for detecting children with overweight and obesity in the prepubertal age were 29 cm and 28 cm for boys and girls, respectively, and in the pubertal period were to be 32.5 cm and 31 cm, respectively, [32]. These findings indicated that all the above-stated indicators can be used for measuring fat deposition in the central or upper portion of the body. However, due to ethnic and geographical disparity, there is still no consensus on which should be used to define obesity and central adiposity for the children and adolescent population. We determined superlative cut-off values of the aforementioned stated anthropometric indicators, for identifying Pakistani children with overweight and obesity. To our best knowledge, this is the first study which was performed among Pakistani children to examine the feasibility and accuracy of these novel indicators simultaneously.

In our study, mean comparison of different anthropometric characteristics (height, weight, NC, WC, and MUAC) by sex indicated that boys had more mean values than those measured for the girls. These results are consistent to earlier studies [16, 33]. Former report also explained that many internal (genetic) and external exogenous factors, growth and nutritional status, food intake pattern, physical activity, etc., may cause the higher anthropometric parameters in favor of boys [3].

For the overall children sample, the AUCs for all proxy indices of detecting childhood obesity ranged from 0.78 to 0.86. For boys, the largest areas were found in AHtR (AUC = 0.860) and WHtR (AUC = 0.855), followed by WC (AUC = 0.805), MUAC (AUC = 0.802), and finally NC (AUC = 0.780). Similarly, the AUC of AHtR (0.825) and WHtR (0.844) also had seen as the largest areas among girls, followed by WC (0.814), MUAC (0.810), and NC (0.780). These findings indicated that while all of these indices performed well in diagnosing obesity, the AUCs for AHtR and WHtR were superior to those for other indicators in both genders. Our study findings were also consistent with earlier studies that showed that AHtR is another highly effective and more reliable screening method for childhood obesity [16, 17].

The diagnostic accuracy for boys vs. girls of the WC (AUC = 0.87 and 0.91 vs. AUC = 0.83 and 0.85; overweight and obesity, respectively), WHtR (AUC = 0.87 and 0.91 vs. AUC = 0.85 and 0.87; overweight and obesity, respectively), waist-to-hip ratio (WHR) (AUC = 0.67 and 0.74 vs. AUC = 0.62 and 0.63; overweight and obesity, respectively), and the NC (AUC = 0.82 and 0.87 vs. AUC = 0.84 and 0.87; overweight and obesity, respectively) also depicted that the WHtR had the outstanding diagnostic achievement of screening childhood obesity among Iranian's [33]. Analyzing the Thai and Chinese school-aged children data, researchers also reported that WHtR is a more accurate predictor of childhood overweight and obesity than WC [17, 34].

The present study results revealed that the cut-off points of AHtR in both sexes were the same (i.e., AHtR = 0.14) for the diagnosing of overweight and obesity, while WHtR cut-off values for overweight and obesity were 0.46 and 0.47 for boys and 0.47 and 0.48 for girls, respectively. Our AHtR cut-offs were nearly similar to the earlier studies for Han children aged 7-12 years (AHtR = 0.15) and for the Thai school-aged children (AHtR = 0.145 for overweight and 0.16 for obesity) [16, 17]. As with AHtR, our WHtR cut-off values were virtually identical to those reported in other research, including 0.45 for overweight and 0.47 for obesity among Thai children [35] and 0.44 for overweight and 0.48 for childhood and adolescent obesity among Chinese aged 8-18 years [34]. This small variation in cut-off values may be explained by the fact that the studies used different definitions of obesity or by the participant age range.

A reliable tool for measuring childhood obesity should meet the following criteria: it should be simple, affordable, simple to use, and agreeable to the participants, while WHtR and AHtR both indicators also have several advantages compared to other indices, e.g., similar to our study (data not shown) for measuring the obesity index. Both of these indices have a pessimistic correlation with age in previous studies [16, 34], allowing for the possibility of age-independent cut-offs (as we did in our study). These cut-offs are easy to manipulate by experts and laypeople alike. These findings may be explained by the fact that both AHtR and WHtR were preadjusted for height, which is highly associated with age [16]. Development is a critical factor in the changing body composition of the pediatric population, and thus, height and age should always be deemed. Overall, we can say that both AHtR and WHtR are inexpensive and easily applicable indices to identify childhood obesity.

We also found that the ability of WC, MUAC, and NC to detect childhood obesity was “highly accurate” (AUC > 0.65) that was consistent with recent findings [10, 14, 15]. However, the diagnostic accuracy of NC was lower than the WC and MUAC. Some other epidemiological researchers also reported that NC is a simple and reliable tool for diagnosing children with a higher BMI; previous findings determined that WC was superior to NC for detecting overweight and obesity [32, 33]. Waist circumference is a highly responsive and precise indicator of abdominal adiposity among children and a good predictor of visceral adiposity. However, WC is not possible in such cases, such as skeletal deformities, intra-abdominal condition, or decrease in abdominal circumference due to respiratory movements. In these settings, MUAC and NC can be used as additional surrogate tests of childhood obesity. Thus, WC, MUAC, and NC can be used as screening methods for childhood and adolescent obesity [10, 15, 17, 33].

The WC cut-off points for obese boys (64.84 cm) and girls (68.29 cm) in our research were lower than the cut-off points for obesity in an Iranian study of children [33]. Additionally, our MUAC cut-off points for diagnosing obesity in boys (18.67 cm) and girls (20.19 cm) were lower than those reported previously [16, 17]. NC 27.05 (sensitivity = 64.3 percent and specificity = 82.4 percent) for boys and NC 27.52 (sensitivity = 67.0 percent and specificity = 75.2 percent) for girls were the most discriminating cut-off values for subjects with obesity (i.e. BMI > 95^th^ percentile). NC cut-off values were found to be significantly higher for the prediction of overweight/obesity in children during the prepubertal (boys vs. girls: 29.0 vs. 28.0) and pubertal (boys vs. girls: 32.5 vs. 31.0) periods [32]. Similarly, much higher cut-off values for NC were found in children aged 6-18 years for the prediction of obesity [10]. As is well established, cut-off values differ across populations, racial groups, and sexes [33]. These factors can account for the greater difference in our cut-off values. Additionally, some research used a different concept of obesity than we do, and this methodological diversity could have an effect on these values [15, 17, 32]. This study's findings have significant clinical and public health consequences. The presented cut-off values for all measures would be especially beneficial for local health care practitioners in identifying and controlling overweight and obese children.

The results of this study may also be used in different fields of interest. For example, several studies have suggested BMI, WC, and WHtR as strong predictors of childhood metabolic risk [36–38]. Savva et al., in a study involving nearly 2,000 children, concluded that WC and WHtR were better predictors of cardiovascular disease risk factors in children than BMI. The authors advised the need for further studies to determine the cut-off points for these indices for an accurate prediction of risk factors [37]. Other studies suggested that indicators such as BMI, WC, and WHtR may be associated with the occurrence of cardiovascular diseases and be useful screening tools for the prediction of these diseases [39, 40]. Nowadays, NC is also a good predictor of cardiovascular risk factors [41]. A study by Androutsos et al. indicated that BMI, NC, WC, and WHtR were correlated with systolic blood pressure, high-density lipoprotein, triglycerides, and insulin-related indices (insulin, homeostasis model assessment, quantitative insulin sensitivity check index, and fasting glucose to insulin ratio) [42]. Therefore, accurate estimation of these indicators could provide clinically useful guidance for physicians to assess chronic disease risks in patients and application of preventative treatments [43].

Although BMI is the traditionally chosen method to evaluate body size in epidemiological studies among the general population, alternative measures such as WC, WHR, or WHtR have been suggested to be superior to BMI in predicting the risk of cardiovascular diseases [44, 45]. Moreno et al. indicated that WC was superior to BMI in predicting metabolic syndrome in children [46], and Valeria et al. [47] showed that WC could predict insulin resistance in children. Moreover, elevated BMI does not always reflect increased adiposity. Individuals with excessive muscle growth can show a high BMI without having an excess of fat and may be misjudged to be obese [48]. A systematic review and meta-analysis of thirty-seven studies that evaluated 53,521 participants from almost all continents showed that BMI has high specificity in identifying pediatric obesity, but moderate sensitivity [49]. As an alternative to other indicators, the NC, MUAC, or AHtR are more practical parameters, which are unaffected by being full or hungry or by respiratory movements and provide more consistent results to indicate body fat accumulation.

Our analysis has a number of advantages. To date, no comparable research has been conducted in Pakistani children to evaluate the predictive output and to determine the optimal cut-off points for various anthropometric indicators used to identify children who are overweight or obese using a large sample. Second, the ROC curve analysis findings from our research are only valid at the national level and probably at the regional level.

Additionally, the analysis has few limitations. Firstly, the causal mechanisms underlying the observed relationships could not be detected in this study due to the cross-sectional nature. Secondly, our proposed cut-offs for different indices for screening obesity may be unreliable for individuals with different health problems like cardiovascular diseases, diabetes, and hypertension.

## 5. Conclusion

Based on ROC analysis findings, we concluded that the anthropometric indicators AHtR, WHtR, MUAC, WC, and NC can be used as screening methods in the assessment of overweight and obesity in children. However, AHtR and WHtR both give the best results for overweight and obesity screening in both sexes. The results suggested that the Pakistani boys and girls, aged 5-12 years with AHtR ≥ 0.14, WHtR ≥ 0.46, MUAC ≥ 18.41 cm, WC ≥ 62.86 cm, and NC ≥ 26.36 cm and AHtR ≥ 0.14, WHtR ≥ 0.47, MUAC ≥ 18.16 cm, WC ≥ 64.39 cm, and NC ≥ 26.54 cm, respectively, could be considered to be overweight. For diagnosing obesity in Pakistani children, the cut-off values for WHtR, AHtR, MUAC, WC, and NC were 0.14, 0.47, 18.67 cm, 64.84 cm, and 27.05 cm in boys and 0.14, 0.49, 20.19, 68.29, and 27.52 in girls, respectively.

## Figures and Tables

**Table 1:** Descriptive statistics for age and anthropometric parameters of the studied children.

Characteristics	Total (*n* = 5,964)	Boys (*n* = 2,865)	Girls (*n* = 3,099)	*p* value
	Mean ± SD Median (IQR)	Mean ± SD Median (IQR)	Mean ± SD Median (IQR)	
Age (years)	8.87 ± 2.36 9.0 (7.0-11.0)	9.23 ± 2.36 10.0 (7.0-11.0)	8.53 ± 2.32 9.0 (6.0-10.0)	<0.001
Height (cm)	130.29 ± 12.95 130.0 (121.0-140.0)	132.78 ± 12.80 134.0 (124.0-142.0)	127.99 ± 12.67 128.0 (119.0-136.0)	<0.001
Body mass (kg)	27.15 ± 7.85 26.0 (21.0-32.0)	28.05 ± 7.71 27.0 (22.0-32.0)	26.31 ± 7.89 25.0 (20.0-31.0)	<0.001
BMI (kg/m^2^)	15.70 ± 2.48 15.26 (14.06-16.91)	15.65 ± 2.42 15.23 (14.09-16.76)	15.74 ± 2.54 15.26 (13.97-17.05)	0.19
NC (cm)	25.30 ± 2.21 25.40 (24.13-26.67)	25.49 ± 2.11 25.49 (24.13-26.67)	25.14 ± 2.29 25.40 (23.37-26.67)	<0.001
WC (cm)	56.89 ± 8.13 55.88 (50.80-60.96)	57.49 ± 8.07 56.39 (52.07-62.23)	56.07 ± 8.09 55.88 (50.80-60.96)	<0.001
WHtR	0.44 ± 0.05 0.44 (0.40-0.47)	0.43 ± 0.05 0.44 (0.40-0.47)	0.44 ± 0.05 0.44 (0.40-0.47)	0.05
MUAC (cm)	17.08 ± 2.32 16.51 (15.24-17.78)	17.23 ± 2.34 16.52 (15.24-17.79)	16.94 ± 2.29 16.51 (15.24-17.78)	<0.001
AHtR	0.13 ± 0.01 0.13 (0.12-0.14)	0.13 ± 0.01 0.12 (0.12-0.13)	0.13 ± 0.01 0.13 (0.12-0.14)	<0.001

IQR: interquartile range; WC: waist circumference; NC: neck circumference; MUAC: midupper arm circumference; BMI: body mass index; AHtR: arm-to-height ratio; WHtR: waist-to-height ratio.

**Table:** (a) Pearson correlation coefficient between body mass index and other proxy indicators of obesity in both sexes and all subjects studied

Anthropometric indicators	Body mass index		Total
	Sex		
	Boys	Girls	
MUAC	0.62^∗^	0.68^∗^	0.65^∗^
AHtR	0.53^∗^	0.54^∗^	0.54^∗^
WC	0.54^∗^	0.59^∗^	0.56^∗^
WHtR	0.43^∗^	0.45^∗^	0.44^∗^
NC	0.53^∗^	0.59^∗^	0.56^∗^

**Table:** (b) Regression analysis for the prediction of body mass index from different anthropometric indicators (i.e., AHtR, WHtR, MUAC, WC, and NC)

	Adj. *R*^2^	*p* value	SE
Model 1	0.506	<0.001	1.74
Model 2	0.517	<0.001	1.72

NC: neck circumference; WC: waist circumference; MUAC: midupper arm circumference; WHtR: waist-to-height ratio; AHtR: arm-to-height ratio; SE: standard error; ^∗^significant values *p* < 0.001; model 1 is a regression model including just anthropometric indicators; model 2 adds age and gender to the predictors of model 1.

**Table 3:** Evaluation of areas under the curve (AUC) for identifying children with overweight and obesity based on different anthropometric indicators.

Anthropometric indicators	Boys (*n* = 2,865)		SE	*p* value	Girls (*n* = 3,099)		SE	*p* value
	AUC	(95% CI)			AUC	(95% CI)		
*Overweight*								
Overall children								
AHtR	0.834	(0.804-0.864)	0.015	<0.001	0.834	(0.811-0.858)	0.012	<0.001
WHtR	0.816	(0.790-0.842)	0.013	<0.001	0.814	(0.789-0.840)	0.013	<0.001
MUAC	0.770	(0.734-0.800)	0.017	<0.001	0.798	(0.771-0.825)	0.014	<0.001
WC	0.762	(0.732-0.792)	0.015	<0.001	0.783	(0.756-0.810)	0.014	<0.001
NC	0.732	(0.700-0.764)	0.016	<0.001	0.741	(0.713-0.770)	0.014	<0.001
Prepubertal children								
AHtR	0.848	(0.809-0.888)	0.020	<0.001	0.857	(0.824-0.891)	0.021	<0.001
WHtR	0.775	(0.729-0.822)	0.024	<0.001	0.788	(0.748-0.829)	0.020	<0.001
MUAC	0.812	(0.767-0.857)	0.023	<0.001	0.825	(0.785-0.866)	0.021	<0.001
WC	0.750	(0.696-0.795)	0.025	<0.001	0.779	(0.739-0.819)	0.021	<0.001
NC	0.743	(0.695-0.792)	0.025	<0.001	0.765	(0.724-0.807)	0.017	<0.001
Pubertal children								
AHtR	0.819	(0.776-0.862)	0.022	<0.001	0.819	(0.786-0.852)	0.017	<0.001
WHtR	0.840	(0.809-0.871)	0.016	<0.001	0.843	(0.810-0.875)	0.016	<0.001
MUAC	0.803	(0.758-0.847)	0.023	<0.001	0.790	(0.755-0.830)	0.020	<0.001
WC	0.830	(0.807-0.867)	0.016	<0.001	0.810	(0.775-0.845)	0.018	<0.001
NC	0.779	(0.741-0.817)	0.019	<0.001	0.761	(0.723-0.800)	0.020	<0.001
*Obesity*								
Overall children								
AHtR	0.860	(0.810-0.900)	0.021	<0.001	0.825	(0.785-0.870)	0.021	<0.001
WHtR	0.855	(0.819-0.891)	0.018	<0.001	0.844	(0.806-0.882)	0.019	<0.001
MUAC	0.802	(0.760-0.850)	0.023	<0.001	0.810	(0.770-0.852)	0.022	<0.001
WC	0.805	(0.763-0.847)	0.021	<0.001	0.814	(0.771-0.857)	0.022	<0.001
NC	0.780	(0.740-0.823)	0.022	<0.001	0.780	(0.735-0.820)	0.014	<0.001
Prepubertal children								
AHtR	0.852	(0.772-0.892)	0.031	<0.001	0.859	(0.781-0.897)	0.030	<0.001
WHtR	0.788	(0.719-0.857)	0.035	<0.001	0.766	(0.698-0.834)	0.035	<0.001
MUAC	0.849	(0.792-0.905)	0.029	<0.001	0.855	(0.796-0.915)	0.030	<0.001
WC	0.814	(0.748-0.880)	0.034	<0.001	0.796	(0.729-0.863)	0.034	<0.001
NC	0.811	(0.754-0.869)	0.029	<0.001	0.810	(0.751-0.869)	0.030	<0.001
Pubertal children								
AHtR	0.866	(0.806-0.926)	0.031	<0.001	0.817	(0.763-0.872)	0.028	<0.001
WHtR	0.903	(0.871-0.936)	0.017	<0.001	0.907	(0.869-0.944)	0.019	<0.001
MUAC	0.841	(0.775-0.906)	0.033	<0.001	0.772	(0.710-0.835)	0.032	<0.001
WC	0.878	(0.835-0.921)	0.022	<0.001	0.850	(0.802-0.898)	0.024	<0.001
NC	0.825	(0.773-0.877)	0.026	<0.001	0.797	(0.737-0.856)	0.030	<0.001

AUC: area under the curve; CI: confidence interval; SE: standard error.

**Table 4:** Suggested cut-off points, sensitivity, and specificity of different anthropometric indicators for identifying overweight and obesity in both boys and girls.

Indicators	Boys (*n* = 2,865)							Girls (*n* = 3,099)						
	Cut-off point	Se (%)	Sp (%)	PPV (%)	NPV (%)	*L*_P_	*L*_N_	Cut-off point	Se (%)	Sp (%)	PPV (%)	NPV (%)	*L*_P_	*L*_N_
*Overweight*													
Overall children													
AHtR	0.14	70.0	88.0	29.28	95.74	6.04	0.33	0.14	70.0	88.6	35.39	94.72	5.86	0.37
WHtR	0.46	73.0	78.0	50.37	95.20	3.27	0.35	0.47	67.0	83.1	46.77	95.00	3.94	0.40
MUAC (cm)	18.41	63.0	83.0	31.31	94.59	3.60	0.45	18.16	65.3	83.4	35.29	94.53	3.92	0.42
WC (cm)	62.86	61.2	80.0	27.67	94.19	3.02	0.49	64.39	55.0	90.5	44.53	93.54	5.78	0.50
NC (cm)	26.36	65.2	73.5	23.73	94.34	2.45	0.47	26.54	59.0	77.8	27.01	93.18	2.66	0.53
Prepubertal children													
AHtR	0.14	81.0	77.4	27.39	95.19	3.59	0.24	0.14	74.2	87.5	23.87	94.95	5.95	0.30
WHtR	0.46	75.2	71.2	47.85	94.65	2.61	0.35	0.48	56.7	90.5	44.89	95.65	5.96	0.48
MUAC (cm)	16.63	71.5	81.1	35.79	95.16	3.78	0.35	16.63	72.5	84.0	37.07	95.90	4.52	0.33
WC (cm)	57.78	57.7	80.4	29.81	92.92	2.93	0.53	60.32	53.4	91.1	32.03	94.12	6.02	0.51
NC (cm)	25.02	70.8	66.3	23.31	94.01	2.10	0.44	24.76	71.3	73.0	26.62	94.91	2.63	0.39
Pubertal children													
AHtR	0.14	69.2	90.2	8.70	89.21	7.08	0.34	0.14	60.5	90.0	50.85	93.87	5.86	0.44
WHtR	0.46	70.8	81.7	8.74	89.26	3.87	0.35	0.48	59.5	91.0	48.94	93.63	4.19	0.35
MUAC (cm)	19.43	74.1	84.6	8.33	89.43	4.82	0.31	19.43	65.0	85.8	40.37	94.32	4.59	0.41
WC (cm)	68.45	65.4	88.6	8.74	89.51	5.74	0.39	64.39	68.5	83.8	38.48	94.75	4.24	0.38
NC (cm)	27.05	69.0	77.0	9.82	89.55	2.99	0.41	27.17	66.5	74.8	28.00	93.80	2.64	0.45
*Obesity*														
Overall children													
AHtR	0.14	79.0	86.0	8.98	96.35	5.60	0.24	0.14	70.6	85.5	17.79	97.43	4.86	0.34
WHtR	0.47	77.0	82.4	10.71	96.20	4.37	0.28	0.49	71.3	89.2	14.01	97.12	6.58	0.32
MUAC (cm)	18.67	73.0	80.3	16.09	96.39	3.70	0.34	20.19	59.0	90.7	10.24	96.95	6.29	0.45
WC (cm)	64.84	73.0	78.0	15.98	95.92	3.26	0.35	68.29	69.1	87.4	13.37	97.34	5.49	0.35
NC (cm)	27.05	64.3	82.4	15.94	96.05	3.65	0.43	27.52	67.0	75.2	8.89	97.33	2.70	0.44
Prepubertal children													
AHtR	0.14	79.1	79.4	14.36	98.16	3.83	0.26	0.14	79.7	81.4	3.26	95.73	4.28	0.25
WHtR	0.46	80.6	68.3	24.19	97.55	2.55	0.29	0.49	57.8	86.9	5.98	96.10	4.41	0.49
MUAC (cm)	16.89	77.6	78.4	19.11	98.15	3.59	0.29	18.16	68.8	92.6	5.27	96.38	9.24	0.33
WC (cm)	60.32	62.7	90.2	29.57	97.34	6.37	0.41	62.99	57.8	93.0	4.83	96.42	8.22	0.45
NC (cm)	25.78	59.7	88.0	24.69	97.07	4.97	0.46	25.27	79.7	73.0	4.42	96.65	2.95	0.28
Pubertal children													
AHtR	0.14	76.3	94.1	3.19	95.47	13.01	0.25	0.14	66.7	87.4	32.09	99.12	5.29	0.38
WHtR	0.48	80.3	88.2	2.59	95.38	6.77	0.23	0.49	83.3	91.4	20.25	98.18	9.73	0.18
MUAC (cm)	20.44	75.0	91.7	2.60	95.66	9.06	0.27	20.70	52.8	92.0	24.36	97.57	6.63	0.51
WC (cm)	66.42	80.3	83.9	4.39	95.74	4.98	0.23	65.78	81.9	81.3	17.56	98.93	4.39	0.22
NC (cm)	27.05	80.3	74.6	5.20	95.83	3.16	0.26	27.17	76.4	71.7	11.58	98.42	2.69	0.33

Se: sensitivity; Sp: specificity; PPV: positive predictive value; NPV: negative predictive value; *L*_P_: likelihood ratio for positive; *L*_N_: likelihood ratio for negative.

## Data Availability

The data used to support the findings of the present study is available in Mendeley (https://data.mendeley.com/datasets/sxgymx5xjm/1). Moreover, it could be requested from the first author Muhammad Asif (Email: asifmalik722@gmail.com).
